# Outcomes of patent foramen ovale greater than 3 mm at birth in extremely low birthweight infants

**DOI:** 10.1186/s12887-023-04119-6

**Published:** 2023-06-15

**Authors:** Sheema Gaffar, Bijan Siassi, Rowena Cayabyab, Mahmood Ebrahimi, Lorayne Barton, Merujan Uzunyan, Rangasamy Ramanathan

**Affiliations:** 1 Division of Neonatology, Department of Pediatrics, Keck School of Medicine, University of Southern California, LAC General Medical Center, 1200 N State St, IRD Building, Room 820, Los Angeles, CA 90033-1029 USA; 2 Division of Cardiology, Department of Pediatrics, Keck School of Medicine, University of Southern California, LAC General Medical Center, Los Angeles, CA USA

**Keywords:** Extremely low birthweight, Flap valve, Neonatal echocardiography, Patent foramen ovale

## Abstract

**Background:**

Foramen ovale (FO) is an obligate fetal shunt that typically resolves after birth, although patency throughout life is not uncommon. The natural history of patent FO (PFO) is known in term infants, but less is known about its course in extremely preterm infants. We describe the echocardiographic changes in FO size from birth to discharge in extremely low birth weight (ELBW) infants in this retrospective study.

**Methods:**

Cohort was divided based on size of FO at birth. Size of FO at discharge was measured and evaluated relative to postnatal weight gain. Demographics and clinical outcomes were compared between the two groups.

**Results:**

Of the 54 ELBW infants, 50 were born with FO less than 3 mm in diameter (small), and 4 were born with FO greater than 3 mm (large). Of small defects, the majority (44/50, 88%) did not increase in size with weight gain, and minority (6/50, 12%) increased in size, and three of these 6 patients, FO grew to be slightly larger than 3 mm. In contrast, all large defects (4 of 4, 100%) nearly doubled in size with postnatal growth. These 4 ELBW infants with enlargement of FO had a flap valve evident on echocardiogram obtained prior to discharge, and subsequently closed on outpatient follow-up echocardiograms, although time to resolution was variable (6 months – 3 years). One infant had presumptive resolution because of the presence of flap valve.

**Conclusion:**

No maternal or neonatal demographic characteristics were predictive of FO enlargement, although, demonstrable flap valve on discharge echocardiogram correlated with resolution of FO on outpatient follow-up echocardiogram. Therefore, based on our data, we recommend that ELBW infants born with large FO should have echocardiographic re-evaluation of the atrial septal opening prior to discharge, to specify the presence of a flap valve or lack thereof, which is an important detail that can help a neonatologist determine the need for outpatient cardiac follow-up.

**Supplementary Information:**

The online version contains supplementary material available at 10.1186/s12887-023-04119-6.

## Background

During the fourth week of embryonic life, the atrial septum begins its separation of the fetal atrium into right and left by formation of septum primum [1]. Subsequently, septum secundum develops to the right of septum primum, overlapping its margins and creating a tunnel-like gap that becomes the FO [2]. In utero, blood is directed from right atrium to left atrium through the FO by a flap valve, bypassing the pulmonary circulation. Derived from the upper portion of septum primum, the flap valve is open by right-to-left (R-L) flow and closed by left-to-right (L-R) flow [3]. The main contributors to FO morphology are degree of septal overlap, which determines tunnel length, and length of flap valve, which determines degree of valvar competence [4].

After birth, the fetal atrial shunt typically resolves with physiologic drop in pulmonary vascular resistance and increase in left atrial blood flow. However, some term infants have minor incompetence of the flap valve, allowing a small L-R shunt that usually resolves by 18 days of life, while a higher percentage of premature infants have persistent valve-incompetent FO which may last for several weeks [5].

Persistence of FO is a well-known occurrence, best highlighted by autopsy of 965 patients that found 80% incidence of PFO at one year of age and 27% in adults [6]. Most of the time, presence of this fetal shunt in postnatal life is clinically insignificant, because left atrial pressure exceeds right atrial pressure, therefore the flap valve will be closed, allowing no shunting through FO [7]. However, with crying and straining in a newborn infant and Valsalva and straining in child and adult, transient R-L shunting can occur [7], posing potential risk for migraine headaches and paradoxical embolism [5, 8, 9], highlighting the importance of identifying the presence of FO. Given that preterm birth and very low birth weight are independent risk factors for overall cardiac morbidity [10, 11], further exploration involving ELBW infants is needed.

It is possible to view FO by 2-dimensional transthoracic echocardiography (ECHO), however, the flap valve is a thin structure in neonates, especially in low birthweight infants. When the flap valve is difficult to image (ECHO “drop out”) [8], color flow Doppler can detect interatrial shunt and serve as a reliable proxy for septal defect size [9, 12, 13].

To our knowledge, this is the first study to describe the echocardiographic change in size of FO in ELBW infants during neonatal intensive care and present evidence for the presence of flap valve predicting closure of FO. We further characterized the outcomes of large FO with postnatal growth of ELBW infants. We hypothesize that PFO size at birth can predict FO size at discharge and that presence of a flap valve predicts FO closure, despite postnatal enlargement.

## Methods

### Study design

Retrospective review was conducted of all inborn ELBW infants (500–999 g) admitted to a tertiary hospital neonatal intensive care unit (NICU) in Los Angeles, California from March 2016 to December 2021 and survived to discharge. Infants were included in the study if an echocardiogram was performed within the first 3 days of life (Echocardiogram 1) and at 36 weeks postmenstrual age (PMA) or within 2 weeks before discharge (Echocardiogram 2). Outpatient follow-up echocardiograms within the first 3 years of life were included for the infants who were followed-up at our institution. Infants with incomplete ECHO series and with significant congenital heart disease were excluded. Institutional review board approval was granted.

### Data collection

Maternal and neonatal demographics were extracted from the neonatal database (Neonatal Information System, NIS5, Medical Data Systems, Rosemont, PA, USA). Data collected from newborn infants included birth weight (BW), gestational age (GA), gender, use of antenatal steroids, APGAR scores, and mode of delivery while maternal demographics included age, self-reported ethnicity, and history of diabetes mellitus, pre-eclampsia and intrauterine infection and/or inflammation. Infant’s PMA and weight at the time of ECHO were also collected. Clinical outcomes of severe intraventricular hemorrhage (IVH) (grade 3 or 4), severe retinopathy of prematurity (ROP) (stage 3 or worse), medical necrotizing enterocolitis (NEC), and bronchopulmonary dysplasia (BPD) requiring oxygen therapy beyond 36 weeks PMA were gathered at the time of discharge.

### Echocardiograms

**Fig. 1:**
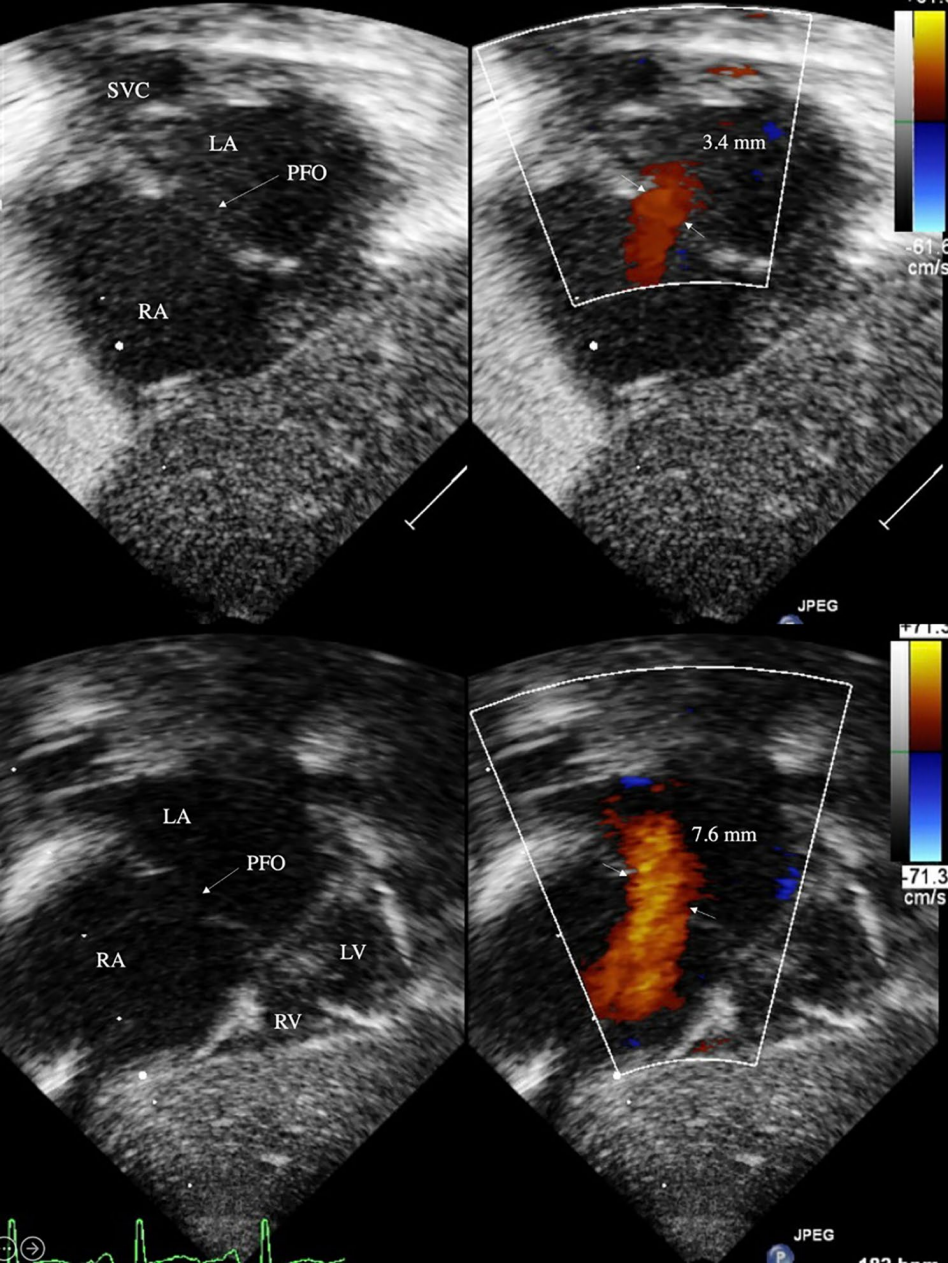
Large FO with L-R shunt at birth (top) and discharge (bottom) in subcostal coronal posterior view

**Fig. 2:**
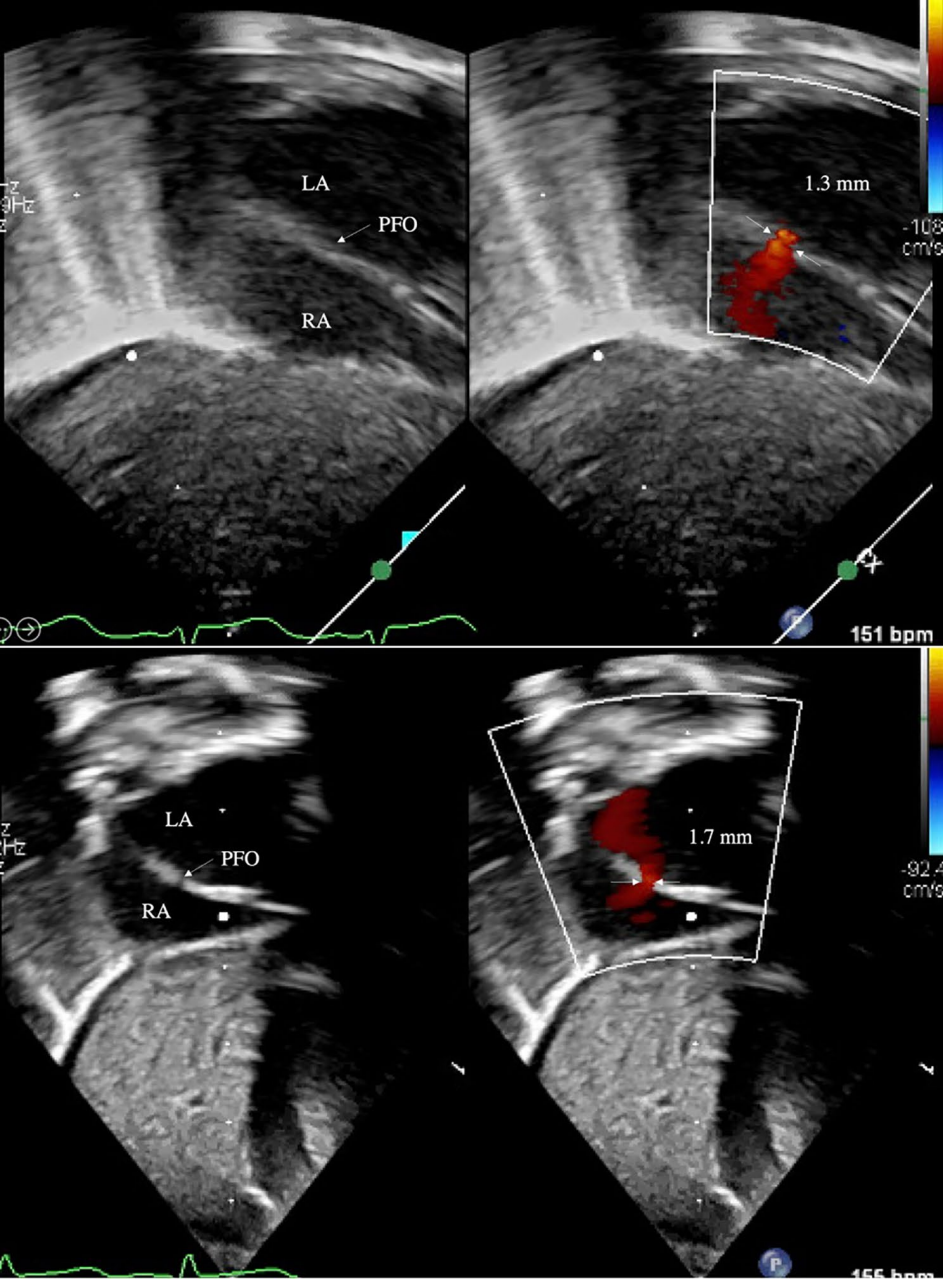
Small FO with L-R shunt at birth (top) and discharge (bottom) in subcostal coronal posterior view

Two-dimensional ECHO series were obtained with Philipps iE33 (Cambridge, MA) and reviewed. Atrial-level shunt was identified in subcostal coronal posterior and subcostal sagittal bicaval acoustic windows, and the region of most laminar flow was located with frame-by-frame advancement (Figs. 1 and 2). Size of valve-incompetent FO was determined by measuring the maximum width of color doppler signal, perpendicular to the direction of flow. In a few patients with very small diameter shunts, where the maximal width of color doppler jet was difficult to identify, 2D measurements were used to obtain the initial FO size. Flow direction and flow pattern across the atrial septum were characterized, along with presence of flap valve in subcostal sagittal view. Previous echocardiographic studies of secundum-type atrial septal defects (ASD) have identified defect size 3 mm as the cusp for congenital heart disease in term infants [14–16]. Therefore, large FO was defined as diameter 3 mm or more and small FO as diameter less than 3 mm. Study cohort of 54 was divided based on FO size on echocardiogram 1, with the hypothesis that ELBW infants with large FO may be at risk for enlargement of the defect with increase in weight. Hemodynamic parameters collected included ejection fraction (EF), fractional shortening (FS), and ratio of left atrium and aorta (LA:Ao). Follow-up echocardiograms were reviewed for size of interatrial defect and presence of flap valve.

### Data analysis

Categorical data was summarized by count and percentage, while continuous variables were represented by median and interquartile range (IQR). Data was further analyzed with bivariate statistics, where categorical variables were compared using Fisher’s exact test and Wilcoxon rank-sum. Stata version 14 (StataCorp, College Station, TX) was used for data analysis. P-value ≤ 0.05 was considered statistically significant.

## Results

### Study population

**Fig. 3:**
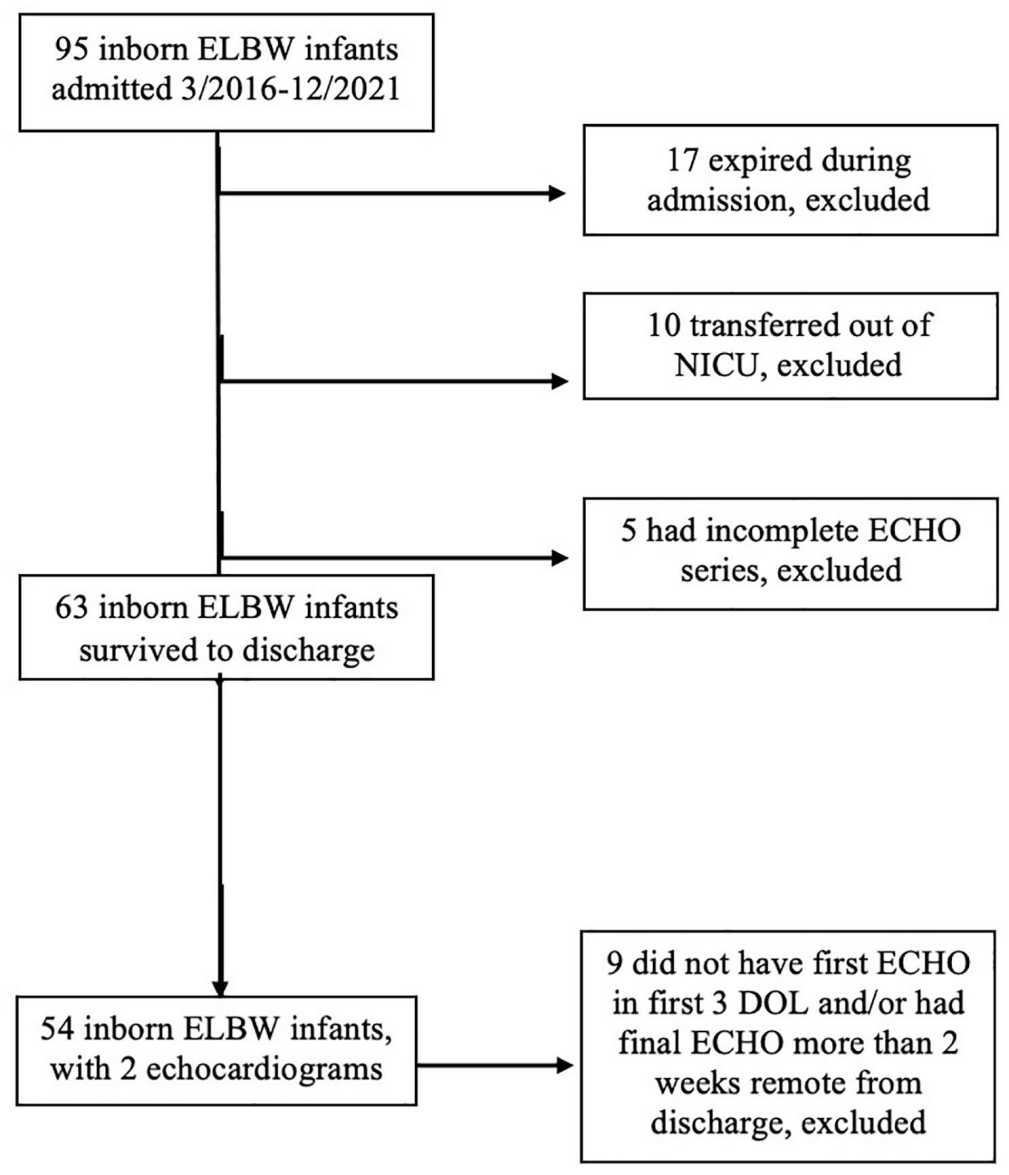
Flow diagram of the study population

There were 95 ELBW infants born from March 2016 to December 2021. Fifty-four infants were included in the study after excluding 41 infants (Fig. 3). Median BW was 768 g (IQR 645, 840) and median GA was 25 weeks (IQR 24, 26). Majority of infants were born to Hispanic women, received antenatal steroids and delivered by cesarean section. Median APGAR scores were 3 and 6 at 1 and 5 min, respectively. The small and large FO cohorts had comparable demographics and clinical outcomes (Table 1).

**Table 1:** Demographic data, echocardiographic data, and clinical outcomes

	Entire cohort (n = 54)	Small FO (n = 50)	Large FO (n = 4)
Neonatal demographics			
Median GA, weeks	25	26	25
Median BW, g	768	778	690
Female sex, %	39	36	75
SGA, %	13	14	0
Cesarean delivery, %	87	90	50
Antenatal steroids, %	56	54	75
Median 1-minute APGAR	3	3	4
Median 5-minute APGAR	6	6	7
Maternal demographics			
Hispanic, %	74	76	50
Black, %	15	16	0
Other ethnicity, %	11	10	25
GDM or T2DM, %	19	20	0
PIH or chronic HTN, %	28	28	25
III, %	6	6	0
Echocardiogram 1			
Median postnatal age, days	2	2	2
FO size^a^, mm	1.9	1.7	3.3*
LA:Ao^a^	11.1	1.1	1.2
FS^a^, %	40	39	42
EF^a^, %	75	75	76
Echocardiogram 2			
Median PMA, weeks	36	36	38
Median weight, g	2303	2300	2590
FO size^a^, mm	2.1	1.9	6.2*
LA:Ao^a^	1.3	1.3	1.3
FS^a^, %	40	40	37
EF^a^, %	74	74	71
Clinical outcomes			
Severe IVH, %	19	16	50
Severe ROP, %	26	26	25
NEC, %	6	6	0
BPD, %	69	68	75
Home oxygen, %	44	46	25
Diuretics, %	24	26	0

*GA* gestational age, *BW* birth weight, *SGA* small for gestational age, *GDM* gestational diabetes mellitus, *T2DM* type 2 diabetes mellitus, *PIH* pregnancy-induced hypertension, *HTN* hypertension, *Triple I* intraamniotic/intrauterine infection, *FO* foramen ovale, *LA* left atrium, *Ao* aorta, *FS* fractional shortening, *EF* ejection fraction, *PMA* postmenstrual age, *IVH* intraventricular hemorrhage, *ROP* retinopathy of prematurity, *NEC* necrotizing enterocolitis, *BPD* bronchopulmonary dysplasia

^a^Calculations done with different denominator than total cohort due to missing data

*Statistically significant (p < 0.01)

### Echocardiograms

**Fig. 4:**
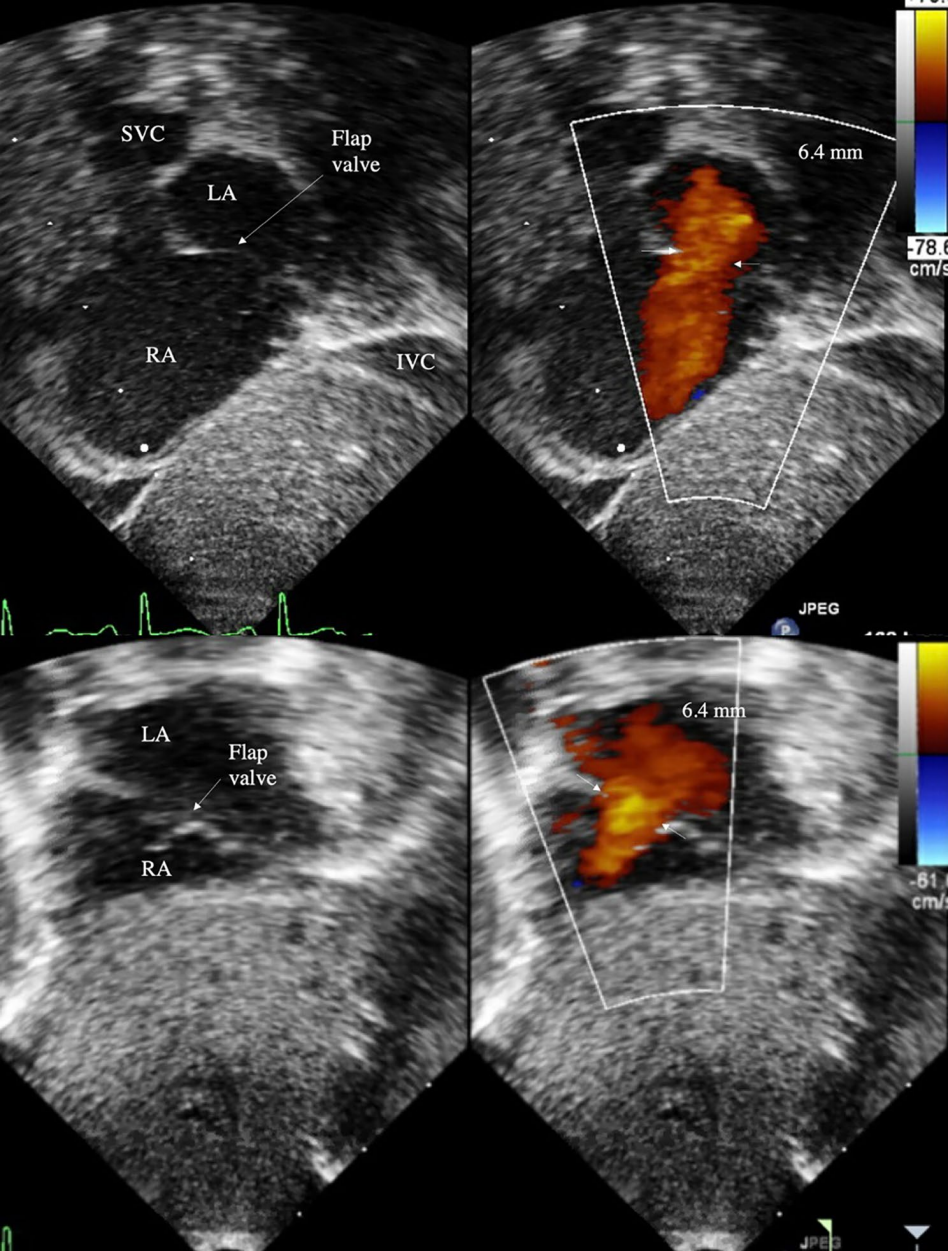
Valve-incompetent FO with large L-R shunt in subcostal sagittal bicaval view (top) and subcostal coronal posterior view (bottom)

At a median postnatal age of 2 days, small FO cohort had a median diameter 1.7 mm (IQR 1.3, 2.3), which was not different at PMA 36 weeks (IQR 35, 39) at 1.9 mm (IQR 1.3, 2.5), despite increase in weight. Of 50 small defects at birth, only 6 had postnatal increase in size, of which only 3 grew to a size greater than 3 mm, with the largest measuring 3.8 mm at discharge. In contrast, 4 infants born with large FO with a median diameter 3.3 mm (IQR 3.1, 3.7) experienced enlargement to 6.2 mm (IQR 5.7, 7.0) at PMA 38 weeks (IQR 36, 42) (p < 0.01). All other echocardiographic parameters were within normal limits for age. Discharge echocardiogram of all 4 infants with large FO had an identifiable flap valve that covered the entire atrial septal defect from the subcostal sagittal view (Fig. 4). Despite postnatal increase in size of 4 large FOs at the time of NICU discharge, 3 defects resolved on outpatient follow-up echocardiogram by age 3 years (Table 2). One large defect that grew from 4.0 to 6.4 mm did not require outpatient cardiology follow-up and was presumed to be resolved due to the presence of flap valve. Interatrial shunt was too small to be measured for 2 patients at birth and 4 patients at discharge, making some data incomplete. Most measured shunts were L-R or bidirectional (95% at birth, 94% at discharge); all were L-R shunts in the large FO cohort.

These results were derived from raw data shown in an additional table [see Additional File 1].

**Table 2:** Follow-up data on 4 ELBW infants born with large FO that initially enlarged with postnatal growth but eventually resolved

	Patient 1^a^	Patient 2^b^	Patient 3^c^	Patient 4^d^
Birth ECHO				
GA, weeks	24	24	26	25
Weight, g	644	760	565	735
FO size, mm	3.4	3.1	3.0	4.0
Discharge ECHO				
GA, weeks	46	39	37	35
Weight, g	4380	2990	2190	3495
FO size, mm	7.5	6.0	5.3	6.4
OP ECHO 1				
GA, weeks	50 weeks	43 weeks	6 months	
Weight, g	4630	3600	6380	
FO size, mm	7.0	5.0	0.0	
OP ECHO 2				
GA, weeks	2 years	55 weeks		
Weight, g	10,400	5900		
FO size, mm	0.0	8.0		
OP ECHO 3				
GA, weeks		1 year		
Weight, g		8205		
FO size, mm		3.0		
OP ECHO 4				
GA, weeks		3 years		
Weight, g		13,400		
FO size, mm		0.0		

*ECHO* echocardiogram, *GA* gestational age, *FO* foramen ovale, *OP* outpatient

^a^Follow-up evaluation for ASD alone

^b^First follow-up evaluation for ASD and PDA. PDA resolved by 4 months corrected age. Subsequent follow-up evaluations for ASD only

^c^First follow-up evaluation for ASD and PDA. ASD resolved by 6 months corrected age

^d^Discharge echocardiogram at 35 weeks was for pre-operative cardiac clearance evaluation

## Discussion

In our study, ELBW infants born with large FO greater than 3 mm had continued enlargement of FO with postnatal growth and weight gain. Flap valve was identified in all of these infants on discharge echocardiogram, potentially predicting the FO closure that was eventually confirmed on outpatient follow-up echocardiogram at 6 months to 3 years of age. In contrast, despite similar somatic growth, all small FO regressed or entirely resolved by NICU discharge. Spontaneous closure of FO has been described [14–20], validating that defects less than 3 mm in diameter do not need a follow up echocardiogram [21].

FO is requisite for in utero development, meaning that all infants have FO patent at birth. While most FO close in the newborn period by way of flap valve, approximately 25–30% of adults have a remnant PFO [22], with its degree of patency associated with increased risk of cardiac and neurologic morbidities [8, 9, 23]. In healthy term infants, the evolution of FO has been well-described both antenatally [24, 25] and postnatally [14–18]. However, studies in preterm infants are sparse and divergent, ranging from case series comparing 187 term and preterm infants [19] to histological study of 30 preterm human fetal hearts at 30–40 weeks’ gestation [26]. As such, it is unknown whether the same morphologic changes that apply to term infants also apply to extremely premature infants.

All ELBW infants in our study had normal cardiac structure and function, with similar hemodynamics as other extremely premature neonates studied previously [14, 27–29]. Except for FO size, there was no difference in echocardiographic characteristics between the two cohorts. Demographics were similar between small and large FO cohorts except for gender, with females representing two-thirds of infants with large FO but only one-third of the overall cohort. This female predilection has been reported in other echocardiographic studies of septal defects in newborns [16] and is consistent with the epidemiology of ASD [30]. In terms of clinical outcomes, there was no difference in IVH, ROP, NEC, and BPD between the cohorts. Contrary to the findings of other studies [20, 31], there was no association between septal defect size and incidence of BPD. While 2 infants with large FO had BPD at the time of discharge, only 1 required home oxygen therapy.

Some institutions perform screening echocardiograms prior to discharge in patients with BPD to evaluate for BPD-associated pulmonary hypertension [32]. Based on our findings, ELBW infants born with large FO should have a repeat echocardiogram prior to discharge to re-evaluate the atrial septum and specifically identify presence or absence of flap valve. While there are no formal guidelines specifying outpatient cardiac follow-up for ELBW infants with a PFO, this approach aligns with recent recommendations of pediatric cardiologists, who recommended repeat echocardiogram in 65–70% of infants with atrial-level shunt 3 mm or greater, and some form of repeat cardiology assessment for 90% of these infants [21]. The study additionally highlighted that while cardiology follow-up was “less frequently recommended for newborns with a patent foramen ovale” [21], additional cardiology follow-up may be desired based on institutional or clinician preference, with greatest variability in timing of follow-up for infants with 3 mm defects across the atrial septum [21].

Transthoracic 2-dimensional echocardiography is a practical tool to image the heart in infants with fair anatomic detail, permitting measurement of width of L-R shunt as a proxy for FO size [9, 14]. With frame-by-frame advancement, shunt across the atrial septum can be examined closely, flow velocity can be measured, direction of flow can be characterized, and comparison can be longitudinal.

Limitations of the study are its retrospective study design, small number of infants with large FO, large number of infants excluded, and lack of generalizability beyond this singular academic center. Echocardiograms have been obtained as part of patient care and specific views to obtain FO size may not be available. Optimal acoustic windows are finite in an agitated or intubated infant. While subcostal coronal posterior view offers the best anatomic perspective of atrial septum to measure interatrial flow, the risk of overestimation of shunt also exists with this orientation [7]. Although the maximal width of color flow doppler shunt across ASD is known to correlate with the size of ASD at surgery [9], pixel number and image resolution are technical aspects that make identification of the maximal width of color flow doppler shunt challenging, especially with small-sized shunts. Limitations of echocardiography, including operator-dependent performance and subjective interpretation, resulted in incomplete data and potentially low interrater reliability.

## Conclusion

ELBW infants born with FO size greater than 3 mm had increase in FO size with postnatal growth. In our examined population, large FO with a flap valve eventually closed, suggesting that presence of flap valve in ELBW infants born with large FO may not need cardiology follow-up after NICU discharge. We therefore recommend re-evaluation of large atrial septal openings with repeat echocardiogram prior to NICU discharge with specific attention to presence of flap valve or lack thereof, which can help a neonatologist determine the need for outpatient cardiac follow-up. Further study of a larger population is needed to elucidate the natural history of atrial septal defects in very premature infants.

## Electronic supplementary material

Below is the link to the electronic supplementary material.

Supplementary Material 1

## Data Availability

All data generated or analyzed during this study are included in this published article and its supplementary information.

## Declarations

**Ethics approval and consent to participate**.

Approval was obtained from the University of Southern California Biomedical Institutional Review Board (IRB), Office of Research and Innovation Human Research Protection Program prior to collection of data (IRB# HS-21-00043). This retrospective chart review study met criteria for exemption as outlined in 45 CFR § 46.104(d). The need for informed consent was waived by the University of Southern California Biomedical Institutional Review Board (IRB), Office of Research and Innovation Human Research Protection Program. This study was performed in accordance with the ethical principles of the Helsinski Declaration and its later amendments or comparable ethical standards. All mothers gave informed consent to have their information gathered and stored in the institution’s clinical warehouse database.

**Consent for publication**.

## Abbreviations

***Ao***: Aorta
***ASD***: Atrial septal defect
***BPD***: Bronchopulmonary dysplasia
***BW***: Birth weight
***ECHO***: Echocardiography
***EF***: Ejection fraction
***ELBW***: Extremely low birth weight
***FO***: Foramen ovale
***FS***: Fractional shortening
***GA***: Gestational age
***GDM***: Gestational diabetes mellitus
***HTN***: Hypertension
***III***: Intraamniotic/intrauterine infection
***IQR***: Interquartile range
***IVH***: Intraventricular hemorrhage
***LA***: Left atrium
***L-R***: Left-to-right
***NEC***: Necrotizing enterocolitis
***NICU***: Neonatal intensive care unit
***OP***: Outpatient
***PFO***: Patent FO
***PIH***: Pregnancy-induced hypertension
***PMA***: Postmenstrual age
***R-L***: Right-to-left
***ROP***: Retinopathy of prematurity
***SGA***: Small for gestational age
***T2DM***: Type 2 diabetes mellitus
